# Development and pilot evaluation of a binocular virtual reality Headset-Based pupillometer for quantitative assessment of pupillary light reflexes

**DOI:** 10.1038/s41598-025-29953-9

**Published:** 2026-01-10

**Authors:** Chan Hae Park, Daseul Kim, Sang Woo Park, Gyuhae Park, Hwan Heo

**Affiliations:** 1 Department of Ophthalmology, Chonnam National University Medical School and Hospital, 42 Jebong-ro, Dong-gu, Gwangju, 61469 South Korea; 2 Department of Mechanical Engineering, Chonnam National University, Gwangju, South Korea

**Keywords:** Pupillometry, Virtual reality, RAPD, Optic neuropathy, Pupillary light reflex, Diseases, Medical research, Neurology, Neuroscience

## Abstract

Relative afferent pupillary defect (RAPD) is a key sign of optic neuropathies but is traditionally assessed subjectively. Virtual reality (VR) headsets with integrated binocular eye tracking enable simultaneous measurement under standardized illumination. We developed and conducted a pilot evaluation of a VR headset–based pupillometer that objectively quantifies RAPD using advanced pupil-tracking and automated blink-correction algorithms. This proof-of-concept study enrolled 17 patients with unilateral optic neuropathy (glaucomatous, traumatic, ischemic, or inflammatory) and 30 healthy controls. A VR headset (FOVE^®^, Tokyo, Japan) recorded binocular pupillary responses at 120 Hz under photopic (~ 130 lx) and scotopic (< 1 lx) conditions. Two protocols captured direct and consensual light reflexes and simulated the swinging flashlight test. Measured parameters included minimum and maximum diameters, constriction percentage, constriction/dilation velocities, and logarithmic RAPD scores. Receiver operating characteristic (ROC) analysis assessed diagnostic accuracy relative to clinical RAPD grading. Patients exhibited significantly reduced direct constriction (26.3% vs. 47.8%; *p* < 0.001), slower dilation velocity, and higher absolute RAPD scores (2.46 ± 2.00 vs. 0.27 ± 0.25 log units; *p* < 0.001) than controls. ROC analysis demonstrated an area under the curve (AUC) of 0.903 for the absolute RAPD score, corresponding to 87% sensitivity and 91% specificity. The VR headset–based pupillometer objectively quantified RAPD by simultaneously recording direct and consensual responses. Given its high diagnostic accuracy, portability, and ease of use, this study provides preliminary evidence supporting the feasibility of VR-based pupillometry as an objective, reproducible alternative to conventional subjective testing for evaluating optic neuropathies.

## Introduction

The pupillary light reflex is an essential neuro-ophthalmic indicator reflecting the integrity of the afferent visual pathways, including the retina and optic nerve, as well as efferent pathways through the oculomotor nerve. Detecting a relative afferent pupillary defect (RAPD) is particularly critical for diagnosing unilateral or asymmetric optic neuropathies, such as glaucoma, traumatic optic neuropathy, ischemic optic neuropathy, and optic neuritis^1^. The conventional swinging flashlight test remains widely used for clinical RAPD detection; however, it is inherently subjective, influenced by ambient lighting, examiner experience, and subtle physiological variations, which can lead to inconsistent results, especially in borderline or early-stage disease^2,3^.

Automated pupillometry has emerged as a promising alternative, providing quantitative and reproducible assessments of pupillary dynamics, thereby minimizing observer-dependent variability inherent in manual testing^4,5^. Nevertheless, many currently available automated pupillometers measure each eye separately, requiring sequential tests and potentially missing subtle interocular asymmetries due to temporal offsets or misalignment. This sequential approach can underestimate consensual reflex differences, limiting diagnostic accuracy. Simultaneously recording both the direct and consensual responses to a single stimulus, as our device does, eliminates this temporal confound, allowing for a more precise and direct comparison of the afferent signal’s integrity^5,6.^

Recently, virtual reality (VR) headsets have gained attention as they directly address the aforementioned limitations of sequential testing. Their ability to provide a fully light-controlled environment while simultaneously tracking both pupils minimizes artifacts from both environmental and temporal variability, respectively^7–9^. While several preliminary studies have demonstrated that VR headset–based pupillometers can reliably detect RAPD^10–12^, a comprehensive validation using robust artifact correction and detailed parametric analysis in diverse clinical populations has been lacking. Furthermore, most existing prototypes have not consistently applied robust blink artifact correction methods, potentially reducing measurement reliability.

In the present study, we aimed to develop and conduct a pilot evaluation of a binocular VR headset-based pupillometer that simultaneously records direct and consensual pupillary reflexes. We hypothesized that this system, with advanced pupil-tracking and automated blink correction, would improve the objective detection and quantification of unilateral optic neuropathy in routine clinical settings.

## Methods

### Study design and participants

This prospective observational study was conducted at Chonnam National University Hospital, a tertiary ophthalmic care center. We enrolled 17 patients diagnosed with unilateral optic neuropathies and 30 healthy controls. Inclusion criteria for healthy controls were best-corrected visual acuity ≥ 20/25, normal ophthalmic examinations, and no previous history of ocular surgery or disease. Exclusion criteria included bilateral optic neuropathy, significant media opacity affecting pupil visualization, efferent pupillary defects, and ocular deviations greater than 30 prism diopters. In patients with unilateral optic neuropathy, the eye with optic neuropathy was designated as the study eye. In healthy controls without interocular differences, one eye was randomly selected as the study eye for analysis purposes. All participants provided informed consent in accordance with the Declaration of Helsinki. Institutional review board approval was obtained from Chonnam National University Hospital (CNUH-2019-260).

### VR pupillometer setup and procedures

All experiments were conducted in a quiet, dedicated examination room with ambient illumination kept below 1 lx. The headset was connected to a standard laptop computer that ran the custom software for stimulus presentation and data recording. We utilized a commercially available VR headset integrated with infrared binocular eye-tracking technology (FOVE^®^, Tokyo, Japan), recording pupillary responses at 120 Hz under standardized photopic illumination (~ 130 lx) and ambient illumination below 1 lx. Although the VR headset provides a largely enclosed visual environment, maintaining ambient illumination below this level minimized stray light leakage through peripheral gaps around the headset and stabilized baseline pupil diameter before each measurement. This setting also ensured comparability with established automated pupillometry protocols conducted under low-light conditions.

Prior to each test session, participants underwent 5 min of dark adaptation to ensure a stable baseline pupil diameter before any light stimuli were presented. Participants were then seated comfortably and instructed to fixate continuously on a central visual target within the VR headset. The fixation target was optically presented at a virtual distance of approximately 6 m, minimizing accommodative and convergent effort and thereby preventing activation of the near triad.

Real-time pupil boundary delineation was performed using advanced image-processing algorithms. This algorithmic approach was adapted from previously validated automated pupillography methods that employed image-based pupil tracking and artifact correction^13^. First, high-contrast grayscale images of the eye were acquired via the integrated 120 Hz infrared cameras. An initial set of candidate pupil boundary points was then identified by applying an intensity threshold to the image, segmenting the dark pupil region from the surrounding iris. To enhance robustness against corneal reflections and other artifacts, geometric constraints were applied to this point cloud. Subsequently, a Delaunay triangulation algorithm constructed a convex hull from the filtered boundary points, providing a robust estimate of the pupil contour even with partial occlusion. Finally, a direct least-squares ellipse fitting algorithm was applied to these contour points to calculate the pupil’s geometric center, major, and minor axes with sub-pixel accuracy, from which the pupil diameter was derived for each frame. Automated blink detection was performed via frame-by-frame threshold-based differencing. Blink-induced gaps lasting less than 1 s were corrected using polynomial interpolation to ensure continuous and stable waveform data. Longer blink-induced gaps prompted immediate repeat testing to maintain data quality.

Two pupillometric examination protocols were conducted sequentially to characterize fundamental pupillary responses and to quantify RAPD, respectively. Real-time direct (stimulated eye) and consensual (fellow eye) pupillary responses were recorded simultaneously. While formal standards for clinical pupillometry are still evolving, the stimulus parameters used in our protocols (photopic illumination of ~ 130 lx for a 3-second duration) are consistent with those used in prior studies to elicit robust pupillary light reflexes for analysis.

**Fig. 1:**
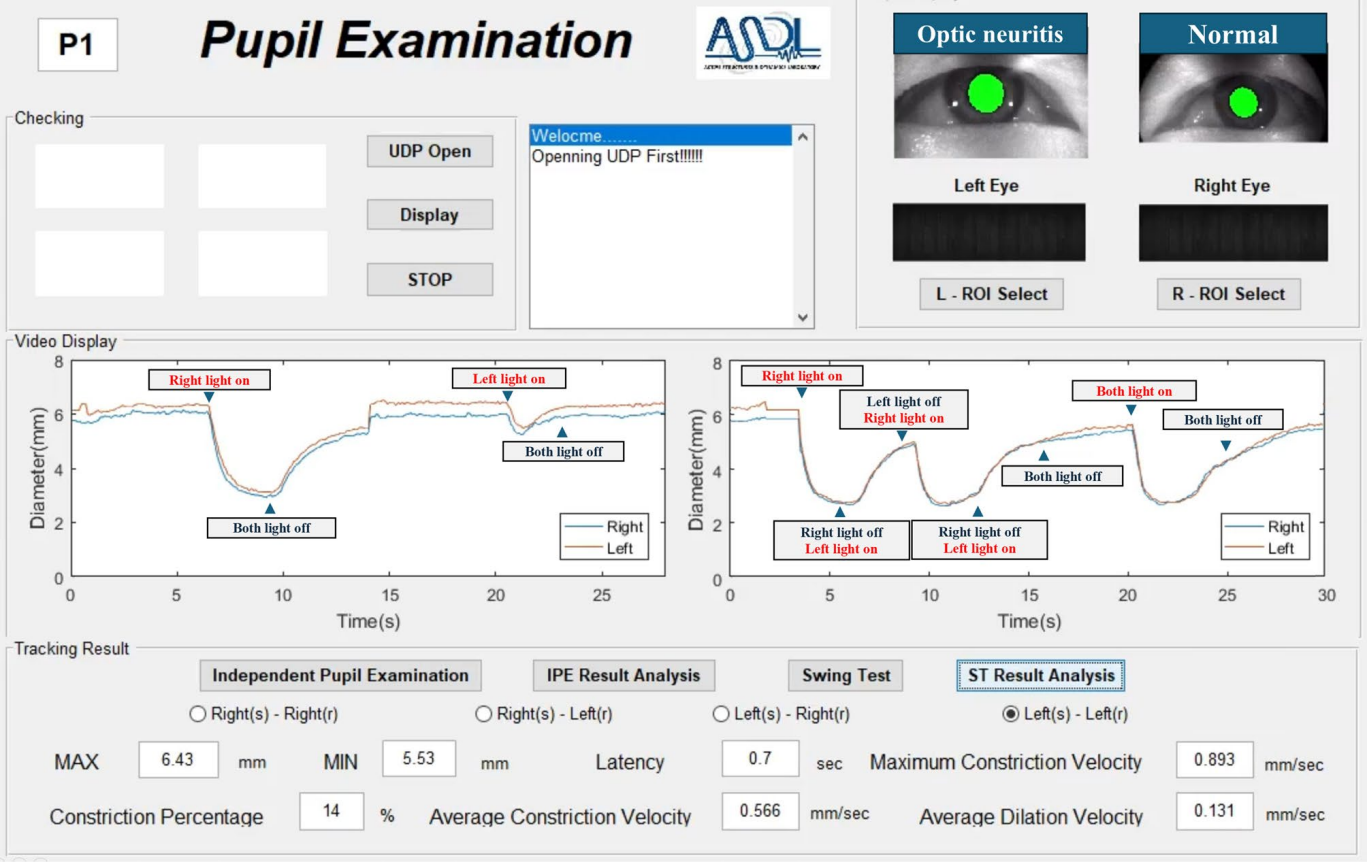
Sample graphical user interface (GUI) illustrating real-time binocular pupillometry protocols, obtained from a representative patient with left-eye RAPD due to optic neuritis; The left panel shows direct and consensual pupillary response measurements, with simultaneous recordings of right (blue trace) and left (orange trace) pupil diameters. The right panel demonstrates the swinging flashlight test protocol, indicating stimulus transitions for assessment of relative afferent pupillary defects (RAPD). Real-time annotations denote stimulus onset and offset. Parameters including maximum/minimum pupil diameters, average constriction velocity, latency, and constriction percentages are displayed and automatically calculated.

**1. Direct and Consensual Pupillary Response** (Fig. 1, Left panel).

- Baseline (Both off): Initial 3 s of darkness.
- Right eye stimulation (“Right light on”): Bright stimulus to the right eye only, lasting 3 s.
- Recovery (Both off): 10 s of darkness.
- Left eye stimulation (“Left light on”): Bright stimulus to the left eye only, lasting 3 s.
- Recovery (Both off): 10 s of darkness.

**2. Swinging Flashlight Test Protocol** (Fig. 1, Right panel).

- Baseline (Both off): Initial 3 s of darkness.
- Right eye stimulation (“Right light on”) for 3 s.
- Switch to Left eye (“Right light off / Left light on”): Immediately after right eye stimulus cessation, stimulus presented to the left eye for 3 s.
- Switch back to Right eye (“Left light off / Right light on”) for 3 s.
- Switch back to Left eye (“Right light off / Left light on”) for 3 s.
- Recovery (Both off) for 6 s.
- Simultaneous Bilateral stimulation (“Both light on”) for 3 s.
- Final recovery (Both off) for 6 s.

This protocol was precisely designed to quantify relative afferent pupillary defects (RAPD). Real-time waveforms, including maximum/minimum pupil diameters, constriction percentage, latency, maximum constriction velocity, and average dilation velocity, were automatically computed and displayed by customized graphical software (Fig. 1).

### Pupillometric parameters (Fig. 2)

**Fig. 2:**
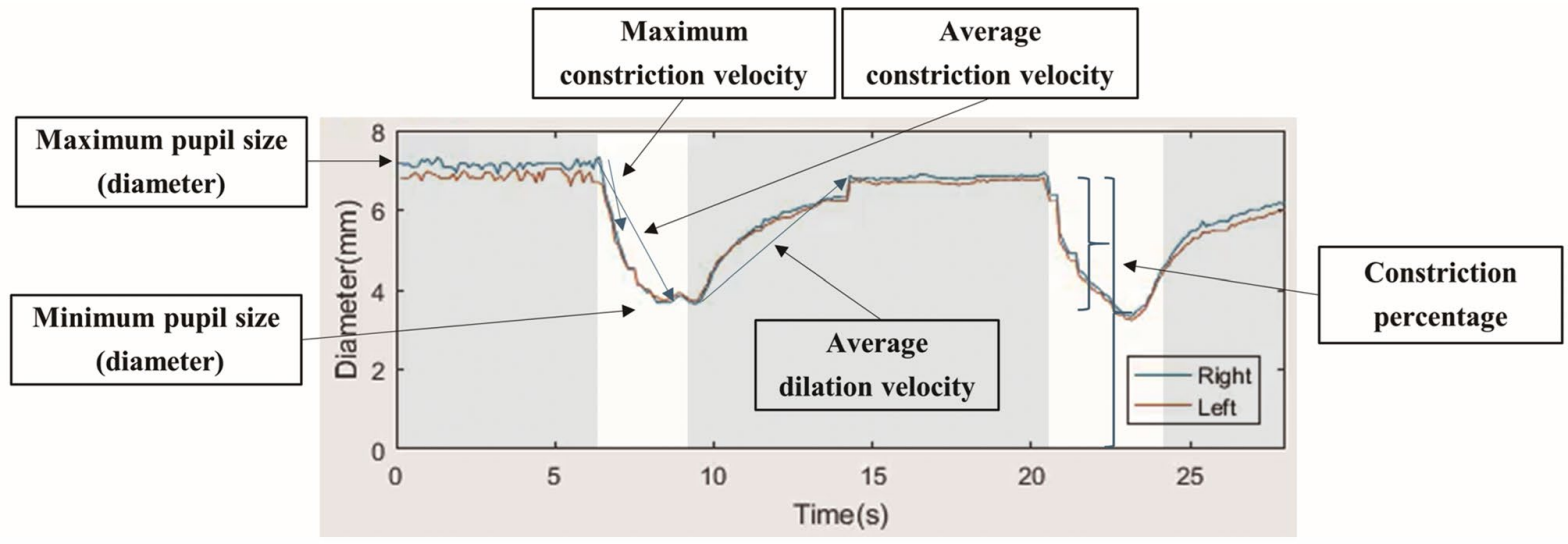
Representative pupillometric waveforms obtained from both eyes (right = blue, left = orange). Parameters include maximum and minimum pupil diameters, constriction percentage (calculated as (Maximum – Minimum)/Maximum × 100), maximum and average constriction velocities, and average dilation velocity. All parameters were automatically calculated by the VR headset-based pupillometer.

The following parameters were automatically calculated for each pupillary response cycle:

- Maximum and minimum pupil diameters (mm).
- Maximum and average constriction velocities (mm/s).
- Average dilation velocity (mm/s).
- Constriction percentage (%), calculated as:

$$\:\text{C}\text{o}\text{n}\text{s}\text{t}\text{r}\text{i}\text{c}\text{t}\text{i}\text{o}\text{n}\:\text{p}\text{e}\text{r}\text{c}\text{e}\text{n}\text{t}\text{a}\text{g}\text{e}\:\left(\text{\%}\right)=\:\frac{\text{M}\text{a}\text{x}\:\text{d}\text{i}\text{a}\text{m}\text{e}\text{t}\text{e}\text{r}\:-\:\text{M}\text{i}\text{n}\:\text{d}\text{i}\text{a}\text{m}\text{e}\text{t}\text{e}\text{r}}{\text{M}\text{a}\text{x}\:\text{d}\text{i}\text{a}\text{m}\text{e}\text{t}\text{e}\text{r}}\:\times\:100$$

- RAPD score (log units, LU), calculated as:

$$\:\text{R}\text{A}\text{P}\text{D}\:\left(\text{L}\text{U}\right)={\text{log}}_{10}\left(\frac{\text{D}\text{i}\text{r}\text{e}\text{c}\text{t}\:\text{a}\text{m}\text{p}\text{l}\text{i}\text{t}\text{u}\text{d}\text{e}\:\left(\text{t}\text{e}\text{s}\text{t}\text{e}\text{d}\:\text{e}\text{y}\text{e}\right)}{\text{C}\text{o}\text{n}\text{s}\text{e}\text{n}\text{s}\text{u}\text{a}\text{l}\:\text{a}\text{m}\text{p}\text{l}\text{i}\text{t}\text{u}\text{d}\text{e}\:\left(\text{f}\text{e}\text{l}\text{l}\text{o}\text{w}\:\text{e}\text{y}\text{e}\right)}\right)$$

The velocity of pupil diameter change was calculated from the first derivative of the pupil diameter data after applying a five-point moving average smoothing filter. Based on this, the following velocity parameters were defined: Maximum constriction velocity - the peak negative velocity (in mm/s) occurring between stimulus onset and the time of minimum pupil diameter. average constriction velocity - the mean of all velocity values calculated between stimulus onset and the time of minimum pupil diameter. Average dilation velocity - the mean of all velocity values calculated from the time of minimum pupil diameter until 2 s after stimulus offset.

A negative RAPD score indicated weaker afferent conduction in the tested eye relative to the fellow eye. Absolute values of RAPD scores were also derived to quantify the magnitude of the deficit objectively^1^.

Absolute RAPD score was defined as the absolute logarithmic ratio of the constriction amplitudes between the two eyes. This value quantifies the magnitude of afferent asymmetry irrespective of laterality (i.e., without indicating which eye is affected), and is particularly used for statistical analyses such as ROC curve evaluation.

### Statistical analysis

Statistical analyses were conducted using SPSS software (version 26.0, IBM Corp., Armonk, NY, USA). Normality of data distribution was assessed using the Shapiro-Wilk test. For normally distributed data, between-group comparisons were made using independent t-tests, with results reported as mean ± standard deviation (SD) and 95% confidence interval (CI). For data that were not normally distributed, the Mann-Whitney U test was used, and results were reported as median and interquartile range (IQR). Effect sizes were calculated where appropriate. A Bonferroni correction was applied to adjust the significance level for multiple comparisons. Receiver operating characteristic (ROC) analysis was performed to evaluate the diagnostic performance of pupillometric parameters, with the area under the curve (AUC) reported with its 95% CI. All statistical tests were two-tailed, and statistical significance was defined as a p-value < 0.05.

## Results

### Demographic data

Seventeen patients with unilateral optic neuropathy (7 glaucomatous, 4 traumatic, 3 ischemic, and 3 inflammatory optic neuropathy) and 30 healthy controls participated in the study. There were no significant differences in mean age between the groups (patients: 48.65 ± 4.66 years vs. controls: 48.00 ± 2.60 years, *p* = 0.904). Sex distribution was comparable between patients (12 males, 5 females) and controls (13 males, 17 females, *p* = 0.071). Visual acuity was significantly poorer in patients (logMAR 1.47 ± 1.13) compared to controls (logMAR 0.03 ± 0.08, *p* < 0.001).

### Direct and consensual pupillary responses

**Fig. 3:**
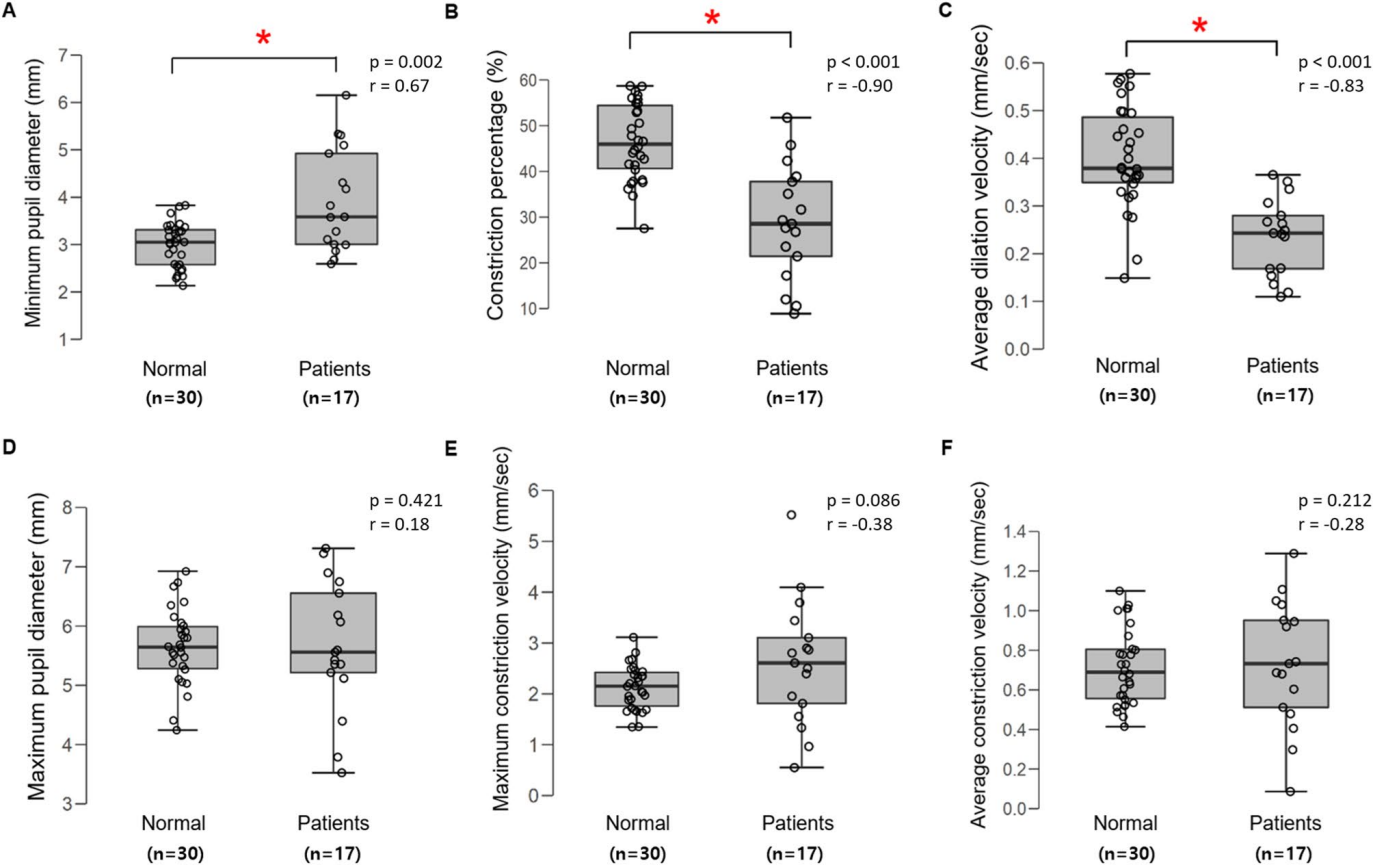
Comparison of pupillometric responses between patients with unilateral optic neuropathy and healthy controls; Significant differences (*p < 0.05) between unilateral optic neuropathy patients and healthy controls were observed for minimum pupil diameter (A), constriction percentage (B), and average dilation velocity (C). No significant differences were found for maximum pupil diameter (D), maximum constriction velocity (E), or average constriction velocity (F).

Patients showed significant impairment in direct pupillary responses compared to controls. Based on the Shapiro-Wilk test, several parameters were not normally distributed. A Mann-Whitney U test indicated that the direct constriction percentage was significantly lower in the patient group (median [IQR] = 26.3% [20.4, 35.7]) than in the control group (median [IQR] = 47.8% [42.1, 51.3]), U = 54, *p* < 0.001. Similarly, minimum pupil diameter was significantly larger (*p* = 0.012, *r* = 0.48) and average dilation velocity was significantly slower in patients (*p* = 0.004, *r* = − 0.61) (Fig. 3A, B, C). However, maximum pupil diameter, maximum constriction velocity, and average constriction velocity did not differ significantly between groups (all *p* > 0.05; Fig. 3D, E, F). Consensual pupillary responses showed no significant differences between patients and controls across all measured parameters (all *p* > 0.05). The presence of intact consensual responses in all participants confirmed that the observed RAPD values were not confounded by efferent pathway abnormalities, thereby ensuring the validity of our measurements. A consistent observation across all measured parameters was the significantly greater variability in the patient group, as shown by the wider interquartile ranges in Fig. 3, which likely reflects the diverse etiologies and severities of optic neuropathy within the cohort.

### RAPD score and Intereye constriction amplitude differences

Absolute values of RAPD scores were markedly higher in the patient group (mean 2.46 ± 2.00 LU, 95% CI [1.48, 3.44]) compared with controls (mean 0.27 ± 0.25 LU, 95% CI [0.18, 0.36]; *p* < 0.001, Cohen’s d = 1.65). Additionally, intereye constriction amplitude differences were significantly greater in patients (1.12 ± 0.94 mm, 95% CI [0.64, 1.59]) than in controls (0.27 ± 0.20 mm, 95% CI [0.20, 0.34]; *p* < 0.001, Cohen’s d = 1.17), indicating pronounced afferent asymmetry in the patient group.

### ROC analysis results

**Fig. 4:**
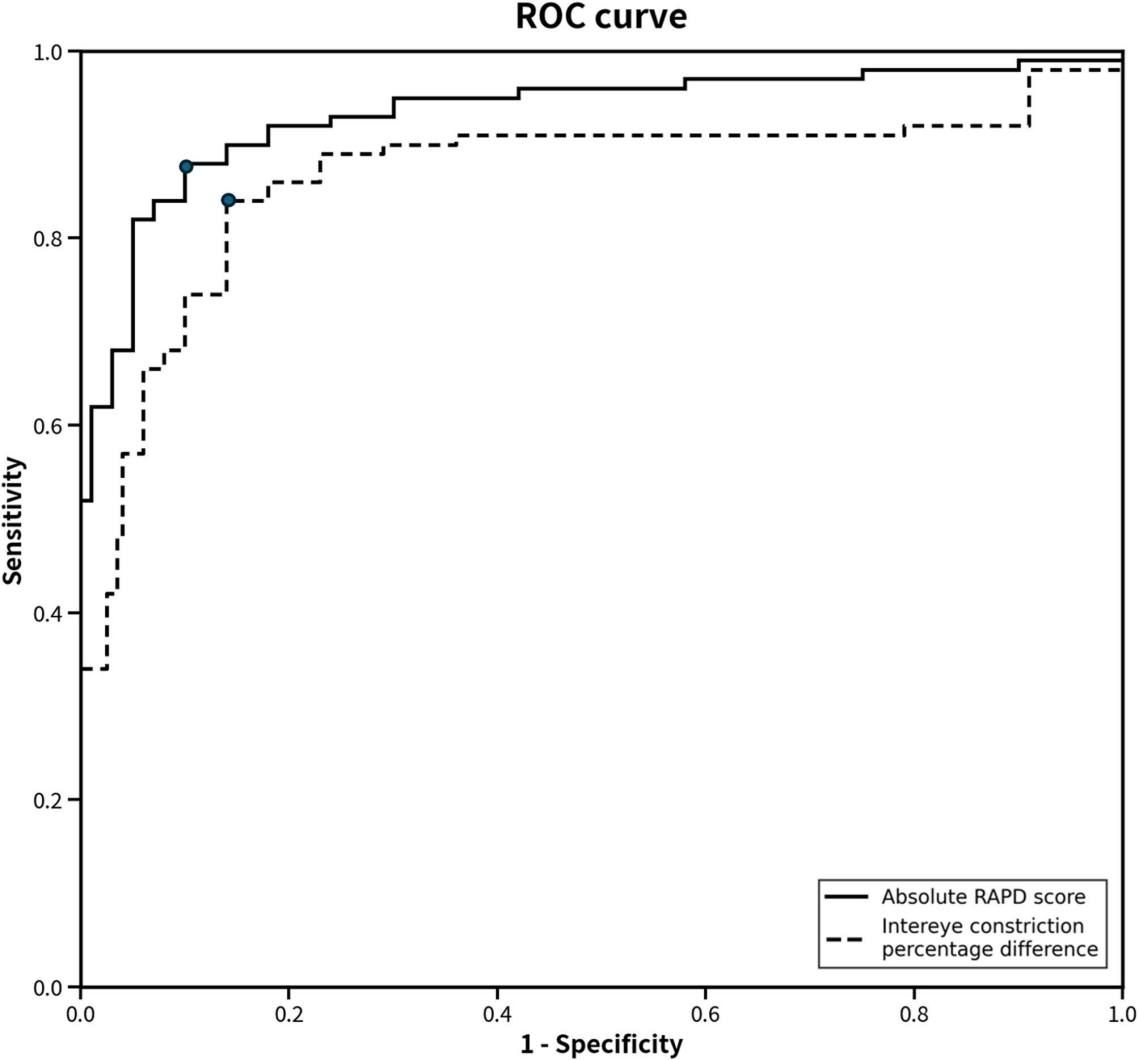
Receiver operating characteristic (ROC) curves for the top two pupillometric parameters for detecting optic neuropathy (17 patients, 30 controls). The absolute RAPD score (solid line; AUC = 0.903, 95% CI: 0.832–0.975) and the intereye constriction percentage difference (dashed line; AUC = 0.811, 95% CI: 0.709–0.912) both demonstrated high diagnostic accuracy. Solid circles on each curve mark the optimal cutoff point as determined by Youden’s index.

To identify the most effective diagnostic parameters, ROC curve analysis was performed (Fig. 4). The absolute RAPD score showed the highest diagnostic performance, with an area under the curve (AUC) of 0.903 (95% CI: 0.832–0.975, *p* < 0.001). At the optimal cutoff point determined by Youden’s index (1.12 log units for the absolute RAPD score), this corresponded to a sensitivity of 87% and a specificity of 91%. The intereye constriction percentage difference also showed good discriminative ability, with an AUC of 0.811 (95% CI: 0.709–0.912, *p* < 0.001). In contrast, parameters based on single-eye measurements, such as constriction percentage and average dilation velocity, showed poor diagnostic accuracy (data not shown). These results indicate that parameters quantifying the relative difference between the two eyes, particularly the absolute RAPD score, were the most effective for distinguishing optic neuropathy patients from healthy controls.

## Discussion

In this pilot study, we developed and validated a novel binocular pupillometer based on a VR headset for the quantitative assessment of pupillary light reflexes. Our results represents a proof-of-concept demonstration of feasibility, showing that the device can effectively distinguish between healthy individuals and patients with optic neuropathy. The capability for simultaneous binocular recording of both direct and consensual pupillary responses is a key feature of this technology. Theoretically, this approach has the potential to reveal inter-ocular differences that might be challenging to capture with sequential monocular assessments, although this hypothesis requires confirmation in future studies specifically designed to test it. Our study provides preliminary evidence that a VR headset equipped with binocular eye-tracking can offer a standardized, objective framework for RAPD quantification.

A principal advantage of our device is its simultaneous binocular measurement capability. Unlike conventional methods or monocular pupillometers that evaluate eyes separately, our VR pupillometer concurrently records both pupils during stimulus presentations, ensuring direct and consensual responses are captured under identical conditions, eliminating variability from separate timing or measurements^14,15^. This approach enables direct comparison of amplitude and kinetics, beneficial for accurately computing metrics such as the RAPD ratio. Previous automated pupillography systems typically employ sequential stimulation, essentially a computerized version of the swinging flashlight test. ^14, 15^ Although effective, these methods still temporally separate the responses of each eye. Conversely, our VR setup continuously monitors both eyes, recording the full waveform of the direct reflex and the consensual reflex simultaneously, potentially improving detection of subtle RAPDs by directly comparing responses in a single trial without mental comparison. Another notable strength is the real-time calculation of multiple pupillary response parameters. Our device instantly computes latencies, velocities, amplitudes, and other quantitative metrics for each stimulus, offering detailed pupillary profiles. For instance, a slowed dilation velocity, as observed in affected eyes, quantitatively demonstrates what clinicians might qualitatively describe as a “sluggish pupil,” thereby facilitating more precise clinical decision-making. Manual assessments typically yield only binary or coarse RAPD judgments and rough severity estimates using neutral density filters, lacking detailed insights into dynamics like velocity or latency^4^.

Objectivity is a major advantage of the VR pupillometer, significantly reducing subjective variability inherent in traditional swinging flashlight tests^2^. Clinicians may differ in their ability to detect subtle RAPDs, leading to inter-observer variability and potential misdiagnoses^2,3^. Even experienced neuro-ophthalmologists may disagree regarding mild RAPD detection. Our automated device provides consistent, unbiased results irrespective of examiner experience, thus improving diagnostic reliability. Moreover, our method expresses RAPD in log units analogous to the neutral density filter grading, simplifying clinical interpretation and integration; for instance, a device-measured RAPD of 0.3 log units directly corresponds to the clinical threshold commonly deemed significant^4^.

Portability and accessibility further distinguish our device. By utilizing commercially available VR headset technology, we significantly reduced costs compared to laboratory-grade pupillography equipment, which typically incurs considerable expense and size limitations. The VR headset-based system is inexpensive, lightweight, requiring only the headset and a laptop. This approach makes it feasible to deploy in diverse clinical settings such as patient bedsides, emergency departments, and primary care offices without the need for specialized dark environments.

The user-friendly design allows broad applicability beyond specialized clinicians. Automated testing procedures require minimal operator training; once the headset is calibrated, even minimally trained technicians can perform testing, expanding usability to general clinical personnel. Potential clinical applications span multiple neuro-ophthalmic and retinal disorders causing asymmetrical afferent deficits. While we focused on unilateral optic neuropathies, conditions like asymmetric macular degeneration or retinal detachment may also produce RAPDs detectable by this device^16,17^. Additionally, glaucoma-related inter-eye asymmetry can yield RAPDs, with automated pupillometry proposed for early glaucoma screening or severity evaluation^6,12^. Moreover, in emergency or neurocritical care environments, standardized automated pupillometry can reduce the variability of neurological assessments.

Our findings are consistent with the broader literature affirming the diagnostic accuracy of automated pupillometry for RAPD detection. Previous portable pupillometers achieved high sensitivity and specificity for glaucoma-related RAPDs,^15^ and VR headset-based pupillometers have recently shown excellent performance in identifying pupillary asymmetries in unilateral optic neuropathies^7,18^. For instance, Bruegger et al. reported a 90.2% sensitivity and 82.2% specificity, with an overall accuracy of 84.4% for RAPD detection using a VR-based headset,^7^ and Negi et al. described performance of a VR-based headset system yielding 85.1% sensitivity and 89.7% specificity in a re-organized ROC analysis accounting for physiological asymmetry^18^. Building on these important findings, our study contributes to this body of work by (1) incorporating an advanced blink-correction algorithm using polynomial interpolation to improve data fidelity, (2) validating the system in a well-characterized cohort with diverse optic neuropathy etiologies, and (3) systematically evaluating the diagnostic performance of multiple kinetic parameters (e.g., constriction/dilation velocities) beyond a simple RAPD score.

Several limitations of this study bear mention. First, the sample size was modest, with only 17 patients with optic neuropathy and 30 healthy controls. Furthermore, the patient group consisted of small subgroups of different etiologies, and the sex distribution between patients and controls was imbalanced. These factors limit the statistical power for subgroup analyses and the generalizability of our findings. Second, and more significantly, our study lacked a stratified analysis based on the severity of optic neuropathy. The diagnostic sensitivity of any RAPD assessment tool is inherently dependent on the clinical spectrum of the patient cohort. Our patient group may have disproportionately included individuals with moderate to advanced optic neuropathy, which could explain the high sensitivity (AUC >0.9) observed for several pupillometric parameters. Consequently, the performance of our device in detecting subtle RAPDs in patients with mild or early-stage optic neuropathy remains to be determined. Therefore, future validation studies should incorporate a larger and more diverse patient population with a well-defined spectrum of disease severities and etiologies. This will be crucial to establish the true diagnostic utility of this technology across a broader clinical context, including in patients with milder optic neuropathies. Furthermore, the VR headset used in this study (FOVE^®^) is a consumer-grade device and not a certified medical device, which presents a barrier to its immediate adoption in routine clinical practice. While our study demonstrates the potential of this approach, further development and regulatory approval would be necessary for clinical translation. It is also noteworthy that other VR-based pupillography systems are emerging for specific clinical applications such as glaucoma management^19^. Additionally, comparing our pupillometer readings, particularly the absolute RAPD score, directly with neutral density filter log units and experienced clinical judgments would further clarify its correlation with established clinical grading. Lastly, patient tolerability, especially in younger or cognitively impaired populations, and the time needed for calibration may influence real-world implementation. Moreover, as this work represents an early pilot investigation, formal test–retest or inter-session reproducibility analysis was not conducted. Because the VR-based pupillometer operates fully automatically without manual measurement, inter-rater variability does not apply. Nonetheless, evaluating both short-term and long-term reproducibility will be essential in future validation studies to confirm the stability and reliability of the measurements across sessions and users. Overall, this work should be regarded as a pilot, proof-of-concept investigation that provides preliminary evidence of feasibility and diagnostic potential. Further large-scale, multicenter studies will be essential to validate and generalize these findings to broader clinical applications.

Nevertheless, this binocular VR headset-based pupillometer objectively and reliably measures pupillary light reflexes and quantifies RAPD, offering a modern alternative to subjective clinical tests. By integrating simultaneous binocular measurements, advanced artifact correction, and real-time output of log-unit RAPD values, this device addresses key limitations of existing systems. With further refinements—such as hardware independence, multi-intensity stimuli, and expanded normative data—it has the potential to become a routine clinical tool that enhances early detection, monitoring, and diagnosis of afferent visual pathway disorders.

In summary, we have developed a binocular VR headset–based pupillometer capable of accurately quantifying pupillary light reflex parameters and RAPD with high diagnostic precision. By integrating simultaneous binocular recordings and advanced artifact correction methods, our system effectively addresses the limitations of sequential testing. Thus, this technology holds significant potential to augment or even replace subjective clinical tests, enabling more reliable detection and management of unilateral optic neuropathies across diverse clinical settings.

## Data Availability

The datasets generated during the current study are available from the corresponding author on reasonable request.
